# Sound Waves Versus Pressure Waves: The Increasing Role of Echocardiography and Noninvasive Vasoreactivity Testing in Pediatric Pulmonary Hypertension Management

**DOI:** 10.1111/echo.70254

**Published:** 2025-07-28

**Authors:** John T. Wren, Kamel Shibbani

**Affiliations:** ^1^ The University of Iowa Stead Family Department of Pediatrics Iowa City USA

Pediatric pulmonary hypertension (PH) is a complex disease with significant morbidity and mortality and affects patients from infancy to adolescence. Although defined simply as an elevation in pulmonary artery pressure (PAP) [1], an understanding of the precise etiology is needed to target appropriate management strategies. This understanding can be derived from Ohm's law, which states that the voltage across two points is a function of flow of electric charge multiplied by resistance (*V* = *I* x *R*). When modified for physiology, this posits that the pressure difference across an organ is a function of the flow of blood into that organ multiplied by resistance (Δ*P* = *Q* x *R*). If the organ of interest is the lung, the formula then becomes PAP ‐ pulmonary capillary wedge pressure (PCWP) = pulmonary vascular resistance (PVR) x pulmonary blood flow (PBF), or when rearranged PAP = (PVR x PBF) + PCWP. Elevated PVR, increased PBF, or increased PCWP can all independently lead to PH. The preponderance of pediatric PH is due to elevated PVR and is also known as precapillary hypertension or pulmonary arterial hypertension (PAH) [2]. However, left to right shunts driving excess PBF or diastolic dysfunction causing elevated PCWP will both raise PAP as well and are frequently encountered in neonates and children [2, 3].

Elucidating these phenotypes and guiding management in pediatric patients requires frequent hemodynamic assessments. The gold standard for this remains right heart catheterization (RHC) and acute vasoreactivity testing (AVT) [2, 4]. However, this procedure is significantly more limited in the pediatric population owing to clinical instability, need for general anesthesia, absence of standardized guidelines, and subspecialty provider availability [5, 6]. In its place, transthoracic echocardiography (TTE) provides a significantly more accessible and noninvasive modality to assess pulmonary hemodynamics [7]. There is a paucity of data, however, regarding the key determination of vasoreactivity responsiveness (and its implications for management and prognostication) in pediatric PH via TTE.

In this edition of *Echocardiography*, Simpkin et al. [8] compare simultaneous TTE‐ and RHC‐based measures of hemodynamics under both baseline and maximal vasodilatory conditions (oxygen and inhaled nitric oxide [iNO]). Although important to benchmark noninvasive metrics with a gold standard, the true value in this study lies in its identification of three noninvasive TTE markers that can predict invasive AVT responsiveness [8]. This has implications for both the neonatal and pediatric populations.

## 1 Neonates

Neonatal PH is a particularly complex disease process owing to the confounding effects of prematurity, rapid changes in PVR, and the presence of shunts. Unfortunately, the diagnostic power of RHC and AVT is often unavailable or not immediately feasible to this population. As a result, assessments of PH are made almost exclusively by TTE. Although this is readily able to estimate pulmonary pressures [7], much less is known regarding noninvasive measures of vasoreactivity in neonates. The emerging field of Neonatal Hemodynamics and neonatologist‐performed targeted neonatal echocardiography (TNE) [9] has provided several insights. In a single‐center retrospective study of preterm neonates born at 22–26 weeks’ gestation with hypoxemic respiratory failure, Boly et al. found that 63% of neonates had a positive response to iNO and this correlated with improved survival (e.g., a noninvasive vasoreactivity “responder”) [10]. Notably, this positive responder status was present in 90% of neonates with TNE‐diagnosed resistance‐mediated PH but was significantly less likely with hypoxemic respiratory failure from other causes (sepsis, patent ductus arteriosus, etc.) [10]. In a population of neonates ≥35 weeks’ gestation with hypoxemic respiratory failure, Bischoff et al. found that while >96% of neonates had PH by TNE, only 63% had a positive response to iNO [11]. Nonresponders were more likely to have additional abnormalities, including impaired left ventricular function [11], suggestive of a non‐PVR‐mediated process.

Although TNE appears to have potential to assess vasoreactivity responsiveness in neonates, its availability is limited to select centers. Fraga et al. performed a single‐center prospective study in infants with bronchopulmonary dysplasia and PH who received iNO and serial TTE [12] and found only a 30% positive responder rate, illustrating the challenges of relying solely on TTE to predict vasoreactivity response. Recognizing the challenge of performing RHC in the highest‐risk neonates, Avitabile et al. aimed to correlate echocardiographic measures of PH with invasive RHC indices (Table 1) in infants with congenital diaphragmatic hernia (CDH) [13]. The investigators found two noninvasive measures that significantly correlated with invasive PAP. Interestingly, in this specific population, only 5% were found to be AVT responders by Sitbon criteria [14]. Noninvasive predictors of RHC AVT response could not be drawn as the echocardiogram was performed a median of 7 days before or after RHC (Table 1) [13].

**TABLE 1:** Findings of relevant studies involving right heart catheterization (RHC), acute vasoreactivity testing (AVT), and transthoracic echocardiography (TTE) in infants and children.

	Study	Median age	Patient population (*n*)	Interval between RHC and TTE	Key findings
Neonates/Infants	Mourani et al. [20]	10.2 months	Chronic lung disease (25)	10.8 days	TTE correctly diagnosed PH by RHC in 79% PAP correlation *r* = 0.19 Estimated sPAP > 40 mmHg by TTE Sensitivity: 0.88 Specificity: 0.33
	Frank, et al. [21]	5.2 months	Premature neonates with BPD (26)	n/a	Reactive Barst AVT response associated with reduced need for tracheostomy or death
	Avitabile et al. [13]	5.1 months	CDH (53)	7.0 days	EIs and RV/LV ratio significantly correlated with RHC PAP (*r* = 0.39 and 0.42, respectively) 5% reactive Barst AVT response rate
Children	Simpkin et al. [8]	9.0 years	Patients with PAH (71)	Simultaneous	Reductions in TTE‐measured TR *V*_max_, *S*/*D* ratio, and EIm predicted Barst AVT responder status PPV: 0.47, 0.47, 0.51, respectively NPV: 0.88, 0.89, 0.78, respectively
	Malakan Rad et al. [19]	3.8 years	Patients with noncyanotic CHD (55)	1.0 days	Four equations identified for echo‐based calculation of sPAP, dPAP, mPAP, and mRAP with a high degree of fidelity
	Sohail et al. [18]	7.5 years	Patients with PAH (50)	Simultaneous	High correlation between sPAP assessed by TTE and RHC Modest correlation between mPAP assessed by TTE and RHC

Abbreviations: BPD, bronchopulmonary dysplasia; CDH, congenital diaphragmatic hernia; EIm, maximal eccentricity index; EIs, systolic eccentricity index; LV, left ventricle; PAP, pulmonary artery pressure; PH, pulmonary hypertension; PPV, positive; RV, right ventricle; S/D ratio, right ventricular systolic to diastolic ratio; sPAP, systolic pulmonary artery pressure; TR *V*_max_, tricuspid regurgitant jet maximum velocity.

**FIGURE 1:**
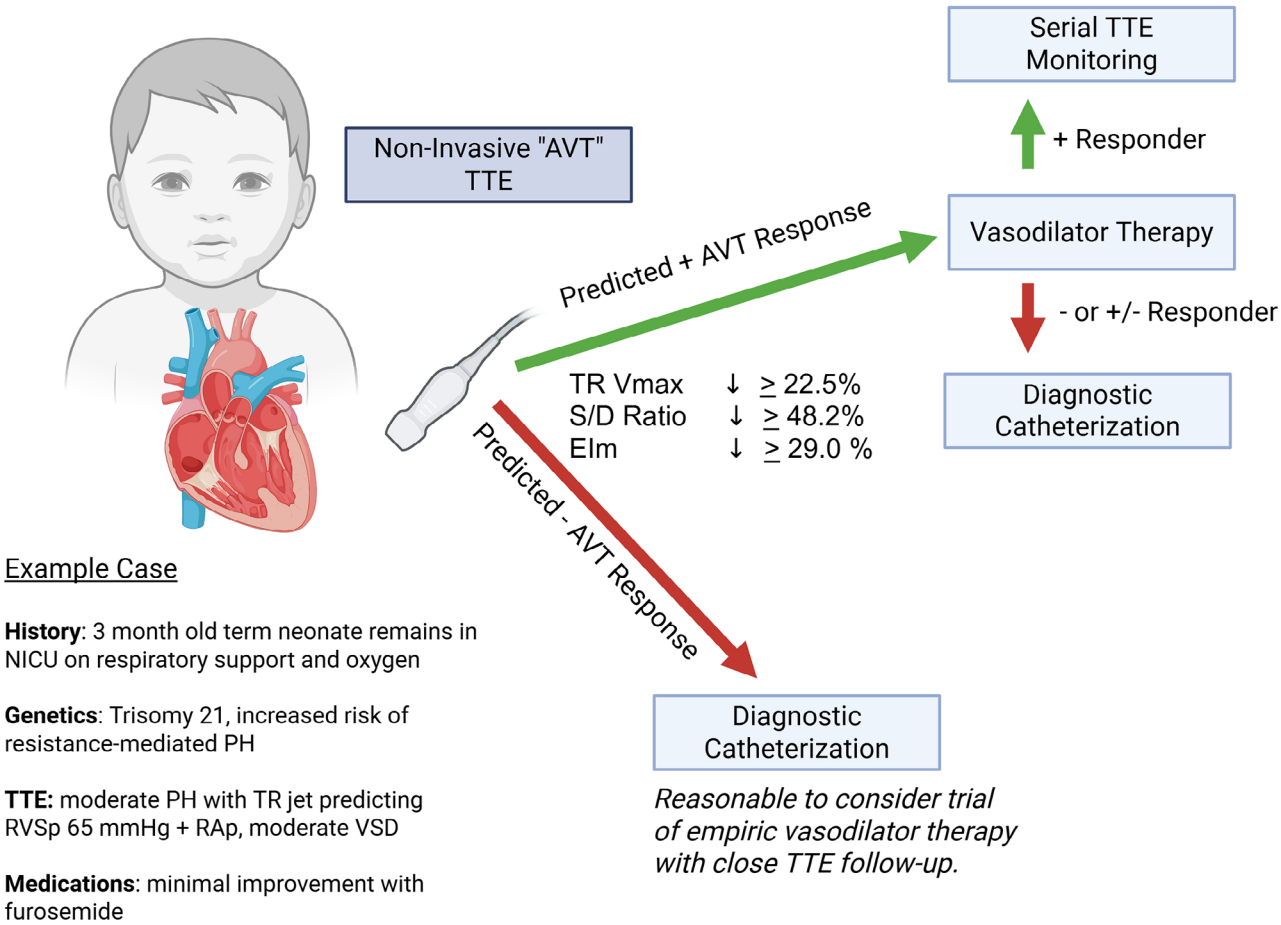
Example case and management algorithm of employing “noninvasive acute vasoreactivity testing (AVT)” to triage need for diagnostic right heart catheterization (RHC). EIm, maximal eccentricity index; NICU, neonatal ICU; PH, pulmonary hypertension; RAp, right atrial pressure; RVSp, right ventricular systolic pressure; *S*/*D* ratio, right ventricular systolic–diastolic ratio; TR jet, tricuspid regurgitant jet; TR *V*_max_, tricuspid regurgitant jet maximum velocity; TTE, transthoracic echocardiography; VSD, ventricular septal defect.

In this study by Simpkins et al., the authors obtain noninvasive TTE hemodynamic indices contemporaneously with invasive RHC measurements [8]. This provides convincing evidence correlating echocardiographic measures with their gold‐standard counterparts. More interestingly, the authors identify three simple‐to‐obtain echocardiographic parameters that predict invasive RHC response (Figure 1) [8]. This has significant implication for the neonatal population, where the ability to obtain RHC is often limited. A predicted positive AVT responsiveness by TTE has the potential to inform the bedside clinician which neonates are most likely to benefit from medications such as iNO, which, when used indiscriminately, have the potential to have no benefit or even to harm [15]. Although a powerful addition to the intensivist's armamentarium, it should not replace the diagnostic precision afforded by RHC and AVT. Instead, this work by Simpkins et al. establishes a triage structure to identify neonates who are prime candidates for vasodilator therapy.

In practice, within the neonatal ICU and Neonatal Hemodynamics program at our center, we will perform a similar “noninvasive vasoreactivity test” via TNE with and without iNO before considering initiation of a long‐term agent such as sildenafil. Those patients with a non‐, negative‐, or un‐clear response are then referred for RHC (depicted as an example case in Figure 1). This approach is supported by a recent cost‐effectiveness and clinical efficacy‐based model as well as consensus guidelines [4, 16]. If validated in neonates, this study would provide greater confidence to employ this RHC triage approach in this vulnerable population. Further work is needed to explore the association of outcomes of RHC AVT responder status in neonates and infants.

## 2 Children

Beyond the benefits of echocardiography‐based markers for assessing AVT in neonates and infants, noninvasively predicting acute vasodilator responsiveness as highlighted by Simpkin et al. is a valuable tool for triaging older children with PH [8]. The authors’ simultaneous acquisition of TTE and RHC data strengthens the clinical relevance of their findings. The high negative predictive value of the identified TTE markers (ranging from 0.78 to 0.89) suggests that a lack of favorable echocardiographic response during vasodilator challenge reliably identifies nonresponders [8]. This can help clinicians avoid unnecessary or potentially harmful therapies in children unlikely to benefit from vasodilators. Conversely, while the positive predictive values of these markers are more modest (0.47–0.51), they still offer a valuable screening tool. In children with suspected PH, a favorable TTE response may justify proceeding with RHC or initiating empiric therapy in settings where catheterization is delayed or unavailable. This approach aligns with emerging models of care that emphasize early identification and stratification of treatment‐responsive therapies [17].

This study also highlights the nuanced relationship between right ventricular (RV) function and pulmonary hemodynamics. Although Tricuspid Annular Plane Systolic Excursion (TAPSE) increased and RV strain rate improved modestly during AVT, other RV function metrics such as the fractional area change (FAC) and strain did not show consistent changes. This underscores the complexity of RV adaptation and PAH and the limitations of relying on a single functional parameter. Nevertheless, the consistent changes in TR *V*_max_, *S*/*D* ratio, and Elm offer a robust and reproducible framework for assessing pulmonary vascular reactivity.

In stark contrast to the adult population, the paucity of research in the noninfant pediatric age group correlating echocardiographic markers with invasive assessment of PH highlights the importance of Simpkin et al.’s study [17]. Sohail et al. assessed the fidelity of echocardiography in estimating systolic, mean, and diastolic pressures when compared with the gold standard of cardiac catheterization [18]. Like Simpkin et al., they found that peak tricuspid regurgitation had the strongest association with direct invasive measurement (correlation coefficient of 0.917) [18]. Malakan Rad et al. assessed the ability to predict systolic‐, mean‐, and diastolic‐PA pressures using novel echocardiographic‐based formulas and found a more modest correlation that varied between 0.7 and 0.8 [19]. PAH in pediatric patients is also confounded by the high prevalence of concomitant congenital heart disease (CHD), with up to 50% pediatric precapillary PH patients having associated CHD [1]. The inclusion of CHD patients in the Simpkin et al. study adds credence to the findings reported.

## 3 Conclusions

The findings of Simpkin et al. support the integration of targeted echocardiographic assessment into algorithms for PH follow‐up. The modest positive predictive value of these findings points to the need for improved markers that can reliably identify response to pulmonary vasodilator management. Although not a replacement for RHC in determining definitive treatment eligibility, echocardiographic assessment during vasodilation challenge can provide actionable insights, especially when invasive testing is limited. Further multicenter validation and longitudinal studies will be essential to confirm these findings and refine their prognostic utility.
